# Supplementary material for: Detection of human disease conditions by single-cell morpho-rheological phenotyping of blood
Source: eLife. 2018 Jan 13;7:e29213. doi: 10.7554/eLife.29213 (PMC5790376; doi:10.7554/eLife.29213)
Supplement: Supplementary file 1. — Percentage of all leukocytes identified by MORE analysis compare to conventional full blood cell counts, obtained with Sysmex XE-5000 differential analyzer and verified by a microscopic differential count, of four donors, two male (A, C), two female (B, D). The absolute cell counts per volume obtained by MORE analysis differ from the values of the conventional blood count, since some cells are not detected (up to 40% of all cells). However, this affects all leukocytes similarly so that the relative counts are not changed. [file elife-29213-supp1.docx]

**SUPPLEMENTARY FILE 1**

|  | Donor A | | Donor B | | Donor C | | Donor D | |
| --- | --- | --- | --- | --- | --- | --- | --- | --- |
|  | MORE^†^ | CBC* | MORE^†^ | CBC* | MORE^†^ | CBC* | MORE^†^ | CBC* |
| neutrophils | 47.2 | 44.2 | 58.9 | 60.1 | 57.0 | 58.1 | 34.7 | 36.5 |
| lymphocytes | 39.4 | 42.2 | 31.3 | 29.7 | 34.3 | 33.5 | 53.3 | 51.0 |
| monocytes | 11.5 | 12.1 | 6.1 | 5.9 | 7.7 | 7.5 | 8.1 | 8.4 |
| eosinophils | 1.2 | 1.4 | 2.9 | 3.2 | 0.5 | 0.6 | 3.4 | 3.6 |
| basophils | 0.6 | 0.1 | 0.8 | 1.1 | 0.4 | 0.3 | 0.6 | 0.5 |

^†^ morpho-rheological (MORE) analysis of whole blood

* conventional blood count (CBC)
