# Supplementary material for: Detection of human disease conditions by single-cell morpho-rheological phenotyping of blood
Source: eLife. 2018 Jan 13;7:e29213. doi: 10.7554/eLife.29213 (PMC5790376; doi:10.7554/eLife.29213)
Supplement: Supplementary file 2. — (1) Morphological analysis of air-dried Romanowsky (Wright, Wright-Giemsa, or May-Grünwald- Giemsa)-stained blood or bone marrow smears. The morphological features identified by microscopic examination may suggest either lymphoid or myeloid differentiation of leukemic cells, but with the exception of the identification of Auer rods in myeloblasts none of these features is lineage-specific. Sub-clones can be identified by differences in size and morphological features (e. g. cytoplasmatic vacuoles). (2) Cytochemical staining improves the accuracy and reproducibility of lineage assessment and therefore is required for traditional sub-classification of acute myeloid leukemia (AML) according to the French-American-British (FAB) and WHO criteria. Sudan Black and stains for myeloperoxidase (MPO) to identify myeloblasts and esterase stains like alpha-naphthyl-butyrate to identify monoblasts have remained useful in this regard. Staining must be performed without undue delay as MPO is unstable and becomes undetectable after a week of storage. (3) Immunophenotypic classification is based on identification of cell surface epitopes or cytoplasmatic proteins by fluorescent dye-labeled antibodies. Flow cytometry (fluorescence-activated cell sorting, FACS) is nowadays widely used as a particularly powerful method because multiparameter analysis offers the advantage of segregating leukemic cells from non-neoplastic cells. Thus, rapid analysis allows to establish the lineage of the leukemia (e.g. myeloid versus lymphoid), its stage of differentiation (e. g. T- versus B-ALL) and facilitates minimal residual disease (MRD) monitoring using a leukemia-specific pattern of markers not expressed in that combination on regular blood or bone marrow cells. Notably, some precursor B-cell ALL might be negative for CD45 (leukocyte common antigen) or patients with T-ALL lack TdT or CD34 expression. Although ALL can be classified according to the stage of maturation, the optimal immunologic sub-cl [file elife-29213-supp2.docx]

**SUPPLEMENTARY FILE 2**

| **Issue of**  **analysis**  **Metho-**  **dology** | *Identifi-cation of leukemic cells* | *Identification of leukemic subclones* | *Classifi-cation of leukemic lineage* | *Lower limit for detection of MRD* | *Amount of material required* | *Time required for ana-lysis* | *Est. cost of ana-lysis*  *[€]* |
| --- | --- | --- | --- | --- | --- | --- | --- |
| ***Morpho-logical analysis*** | Yes | Only if based on morpho-logical differ-rences | Uncertain | 1 / 1x10^2^ | 100 µl | 1 – 2 h | 5 |
| ***Cyto-chemical***  ***staining*** | Yes | Yes | Yes | 1 / 1x10^2^ | 100 µl | 1 –2 h | 10 |
| ***FACS analysis*** | Yes | Yes | Yes | 1 / 5x10^3^  – 1x10^4^ | 100 – 1000 µl | 1 – 4 h | 100 - 500 |
| ***Molecular analysis*** | Yes | Yes | Yes | 1/ 1x10^4^  – 1x10^6^ | 5 – 10 ml | 1 – 10 days | 500 - 5000 |
| ***MORE analysis*** | Yes | Yes | Possible, but not yet formally tested | Possible, but not yet formally tested | 10 µl | 30 min | 20 |
